# Phylogenomic history of enigmatic pygmy perches: implications for biogeography, taxonomy and conservation

**DOI:** 10.1098/rsos.172125

**Published:** 2018-06-13

**Authors:** Sean J. Buckley, Fabricius M. C. B. Domingos, Catherine R. M. Attard, Chris J. Brauer, Jonathan Sandoval-Castillo, Ryan Lodge, Peter J. Unmack, Luciano B. Beheregaray

**Affiliations:** 1 Molecular Ecology Laboratory, College of Science and Engineering, Flinders University, GPO Box 2100, Adelaide, South Australia 5001, Australia; 2 Instituto de Ciências Biológicas e da Saúde, Universidade Federal de Mato Grosso, Pontal do Araguaia, MT 78698-000, Brazil; 3 Institute for Applied Ecology, University of Canberra, Canberra, Australian Capital Territory 2601, Australia

**Keywords:** cryptic species, ddRAD-seq, freshwater fish, historical biogeography, phylogeography, *Nannoperca*

## Abstract

Pygmy perches (Percichthyidae) are a group of poorly dispersing freshwater fishes that have a puzzling biogeographic disjunction across southern Australia. Current understanding of pygmy perch phylogenetic relationships suggests past east–west migrations across a vast expanse of now arid habitat in central southern Australia, a region lacking contemporary rivers. Pygmy perches also represent a threatened group with confusing taxonomy and potentially cryptic species diversity. Here, we present the first study of the evolutionary history of pygmy perches based on genome-wide information. Data from 13 991 ddRAD loci and a concatenated sequence of 1 075 734 bp were generated for all currently described and potentially cryptic species. Phylogenetic relationships, biogeographic history and cryptic diversification were inferred using a framework that combines phylogenomics, species delimitation and estimation of divergence times. The genome-wide phylogeny clarified the biogeographic history of pygmy perches, demonstrating multiple east–west events of divergence within the group across the Australian continent. These results also resolved discordance between nuclear and mitochondrial data from a previous study. In addition, we propose three cryptic species within a southwestern species complex. The finding of potentially new species demonstrates that pygmy perches may be even more susceptible to ecological and demographic threats than previously thought. Our results have substantial implications for improving conservation legislation of pygmy perch lineages, especially in southwestern Western Australia.

## 1. Introduction

The biogeographic histories of species contain information about their past distribution and evolutionary trajectories that can clarify key aspects about community assemblages, biotic exchanges and environmental determinants of biodiversity [1–4]. In fact, coherent patterns of distribution and evolutionary history are often identified in analyses of multiple codistributed taxa, which points to commonalities in biogeographic history [5–7]. On the other hand, biogeographic distributions that cannot be related to ecology or explained by dispersal or vicariance offer enigmatic puzzles that require additional investigation (e.g. [8,9]). Biogeographic knowledge can be further used in applied research, such as assessments of taxonomy and improving conservation legislation [10]. Species delimitation, for example, is a type of phylogenetic analysis that uses computational modelling to determine the number of putative species in a group by using coalescent methods [11,12]. This can lead to revisions of taxonomic uncertainties, a critical aspect of conservation legislation. For example, the identification of independently evolving but cryptic lineages within a single threatened taxon indicates that conservation strategies should manage these lineages separately and avoid outbreeding depression and hybridization among lineages [13].

With the expansion of next-generation sequencing (NGS) technologies, the ability to sequence a representative subset of the genome or even full genomes has become a feasible process in phylogenetics [14–17]. It allows analyses of thousands or more DNA markers, providing high power for circumventing locus-specific biases and inferring phylogenies. Furthermore, no *a priori* genomic resources are required [18–20]. One popular method for obtaining genomic data for non-model species is restriction site-associated DNA sequencing (RAD-seq), which uses restriction enzymes to break down the genome to easily sequenced fragments [21] that can be reconstructed into thousands of DNA loci for estimating robust phylogenetic trees [22,23]. The applicability of RAD-seq in phylogenetics has been recently assessed, with overall support for the method, especially for studies involving recently diverged taxa and complex phylogenies [20,24–26].

Phylogenomics can be applied to complex and conflicting biogeographic assessments in a more robust manner than traditional phylogenetic analyses. One long-standing biogeographic puzzle to be addressed using phylogenomics relates to the currently disjunct distribution of populations and closely related lineages of several aquatic-dependent taxa across southern Australia [27,28]. These taxa, which include crustaceans, fishes and frogs, occur in a distinct temperate zone in the west (i.e. southwest Western Australia) and the east (i.e. the Murray–Darling Basin and southeast coast) of southern Australia, which are isolated by a vast expanse of arid habitat and the limestone-rich Nullarbor Plain in central southern Australia. The palaeoclimate of the area is relatively well-understood: the aridity of central southern Australia developed starting in the Oligocene [29,30] followed by the formation of the Nullarbor Plain 14–16 Ma in the mid-Miocene [27,31]. By the mid-Miocene aridity was firmly established based on the lack of connected drainages across the Nullarbor Plain [27]. Given this, as one would parsimoniously expect, aquatic-dependent lineages on either side of the plain typically show reciprocal monophyly based on genetic data [28,29,32]. However, there are exceptions to this pattern, such as that observed in pygmy perches (Teleostei, Percichthyidae) [27].

**Figure 1.**
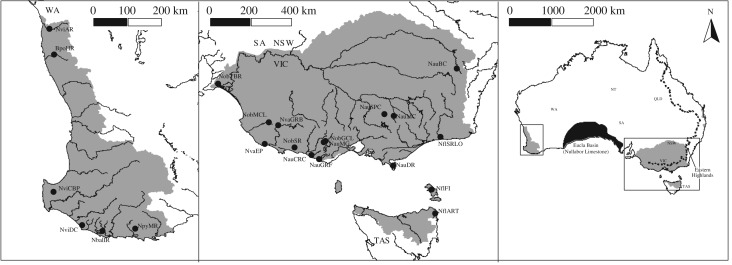
Contemporary distributions of pygmy perch species and populations used in this study. Population abbreviations are denoted within table 1. Population locations are not shown for *N. oxleyana*, which occupies a small region of lower Queensland (distribution indicated in the right figure). The Nullarbor Plain barrier is indicated by the black section of the right figure.

Pygmy perches are composed of species endemic to the southeast (e.g. Murray–Darling Basin and coastal drainages) and the southwest (figure 1): six recognized species within *Nannoperca*, namely *N. australis*, *N. obscura*, *N. oxleyana*, *N. pygmaea*, *N. variegata*, and *N. vittata* [27,33,34], and the sole member of *Nannatherina*, *Nth. balstoni*. A previous phylogeny of pygmy perches supported multiple divergences or east–west migrations prior to the formation of the Nullarbor divide [27]. That study used limited genetic data (the cytochrome *b* gene, three nuclear loci, and allozymes), which could limit their ability to determine accurate inferences of species relationships. It is further compounded by the discordance of phylogenetic trees across the molecular markers used, which showed contrasting phylogenetic relationships both within and across marker types. For instance, there were unresolved phylogenetic relationships between *N. australis*, *N. obscura*, *N. oxleyana* and *N. variegata*, possibly resulting from the complete mitochondrial introgression of *N. obscura* by *N. australis* [27,35].

**Table 1.** Locality data for all species and individuals examined. Population abbreviations described in the table were those used for further analyses, while *n* refers to the number of individuals sequenced per locality.

species	location (*n*)	population abbreviation	field code
*Bostockia porosa*	Hill River (2)	BpoHR	F-FISHY6:DM184+
	Canebrake Pool (1)	BpoCBP	F-FISHx2:P21+
*Nannatherina balstoni*	Inlet River (2)	NbalIR	F-FISHx2:B6+
*Nannoperca variegata*	Glenelg River (2)	NvaGRB	PU00-15VPP
	Ewen Ponds (2)	NvaEP	F-FISH83:HS63+
*Nannoperca vittata* sp. 1	Arrowsmith River (2)	NviAR	F-FISHY6:DM150+
*Nannoperca vittata* sp. 2	Doggerup Creek (2)	NviDC	PU09-49NV
*Nannoperca vittata* sp. 3	Canebreak Pool (2)	NviCBP	PU09-58NV
*Nannoperca pygmaea*	Mitchell River (2)	NpyMR	PU09-37
*Nannoperca oxleyana*	Stradbroke Island (4)	NoxSI	F-FISH93:BF1+, CF1+
*Nannoperca obscura*	Gnarkeet Creek (2)	NobGCL	PU00-27YPP
	Shaw River (2)	NobSR	PU02-113YPP
	Tookyaerta [breeders] (2)	NobTBR	YPBr+
	Mosquito Creek (2)	NobMCL	PU00-16YPP
*Nannoperca australis*	Darby River (2)	NauDR	PU02-70SPP
	Gellibrand River (1)	NauGRF	PU02-92SPP
	Mundy Gully (1)	NauMG	SPP08-11
	Curdies River (1)	NauCRC	PU00-24SPP
	Swanpool Creek (1)	NauSPC	PU09-03SPP
	Blakney Creek (2)	NauBC	F-FISH98:LPP-3+
	Meadows Creek (2)	NauMC	rCB1301+
*Nannoperca* ‘flindersi’	Snowy River (2)	NflSRLO	PU99-85SPP
	Anson River (2)	NflART	F-FISH82:HT-20+
	Flinders Island (2)	NflFI	F-FISH84:FI-3+

Issues with the pygmy perch phylogeny also extend to taxonomy, with some studies suggesting there are cryptic species in *Nannoperca*. Within *N. australis*, populations from the eastern portion of its range have been suggested as a separate species informally referred to as *N.* ‘flindersi’ that is awaiting formal taxonomic review [27,36]. Within *N. vittata*, Unmack *et al.* [27] suggested that a second cryptic species was present. Additionally, *N. pygmaea* was only recently formally described as a distinct species from its putative sister species *N. vittata* based on the morphological and allozyme assessment by Morgan *et al.* [34]. Most pygmy perch species are currently threatened or endangered, thus confirming their taxonomy and identifying cryptic species is imperative for well-informed conservation. Five species (*N. obscura*, *N. oxleyana*, *N. pygmaea*, *N. variegata* and *Nth. balstoni*) are listed as threatened in national legislation [33], all species excluding *N. vittata* are listed in their respective state legislation, and several species have also been listed from vulnerable to endangered in the International Union for Conservation of Nature (IUCN) Red List (IUCN, 2015). Declines in populations of these species have been linked largely to habitat degradation as a result of human-altered hydrological conditions (such as damming and agricultural irrigation) and competition or predation by introduced fish species [27,33,37–40]. These pressures are expected to be exacerbated by a drying climate due to contemporary climate change, with large impacts on ecologically specialized species or those with limited distributions, such as pygmy perches [33,38,39,41].

Here, we capitalize on NGS technology to address uncertainties in the evolutionary history of pygmy perches, to assess the taxonomic identity of recently suggested or described species, and to investigate the possibility of further cryptic diversity within pygmy perch lineages. We present phylogenetic trees based on genome-wide data and use a species delimitation framework and divergence estimates encompassing samples from all described species of pygmy perches. Our findings have implications for clarifying the puzzling biogeography of southern Australia and for the taxonomy and conservation management of a threatened group of endemic freshwater fishes.

## 2. Material and methods

### 2.1. Sample collection and ddRAD library preparation

Specimens were selected to represent the breadth of currently described pygmy perch species, totalling 45 individual samples across seven formally recognized species and all known potential cryptic forms (table 1). Our samples include populations spanning each species range and representatives of all known lineages, including known evolutionarily significant units (ESUs) within each taxon [27,36,39,40,42] (figure 1). The most closely related extant sister lineage to pygmy perches, the nightfish *Bostockia porosa* was included as the outgroup [27]. Specimens were collected using electrofishing, dip-, fyke- or seine-netting. Either the caudal fin or the entire specimen was stored at −80°C at the South Australian Museum, or in 99% ethanol at Flinders University.

DNA was extracted from muscle tissue or fin clips using a modified salting-out method [43] or a Qiagen DNeasy kit (Qiagen Inc., Valencia, CA, USA). Genomic DNA was checked for quality using a spectrophotometer (NanoDrop, Thermo Scientific), integrity using 2% agarose gels, and quantity using a fluorometer (Qubit, Life Technologies). The ddRAD genomic libraries were prepared for the 45 samples in house following Peterson *et al*. [44], with modifications as described in Brauer *et al.* [39]. Genomic libraries were paired-end sequenced on an Illumina HiSeq 2000 at Genome Quebec (Montreal, Canada).

### 2.2. Sequence filtering and alignment

The resultant reads were filtered and cleaned to create a set of aligned concatenated sequences for statistical analyses. The raw sequences were demultiplexed using the ‘process_radtags’ module of STACKS 1.29 [45], allowing up to 2 mismatches in the 6 bp barcodes. Barcodes were removed and sequences trimmed to 80 bp to remove low-quality bases from the end of the reads. These cut reads were then aligned using the software package PyRAD 3.0.6 [46], and further cleaned by removing reads that had more than 5 bp with a PHRED score of less than 20. As PyRAD is a de novo assembly pipeline, it is particularly effective for large-scale, divergent sequences which may cause some sequences to ‘drop out’ due to indels or mutations [46].

To account for the effect of proportions of missing data in the alignment on downstream analyses [20,47], two alignment criteria were used based on minimum coverage: a strict dataset with a minimum coverage of 40 individuals per locus (approx. 89%), and a relaxed dataset with a minimum coverage of 32 individuals per locus (approx. 70%).

### 2.3. Sequence divergence analysis

The genetic distance between concatenated ddRAD sequences from different individuals was calculated using an uncorrected (*P*) distance matrix generated in PAUP* 4 [48], with mean values across lineages (species and, in the case of *N. vittata* and *N. pygmaea*, geographical groups and pairs of lineages). This was done for each filtered dataset (strict and relaxed) to compare the effects of the filtration process on downstream analyses.

### 2.4. Phylogenetic analysis

In order to determine evolutionary relationships among pygmy perch samples, maximum-likelihood (ML) phylogenies were estimated using RAxML for both the strict (4381 loci) and relaxed (13 991 loci) dataset [49]. RAxML remains one of the best options available to estimate a ML phylogeny in genome-wide datasets [49]. This was done using rapid hill-climbing and 1000 resampling estimated log-likelihood [50] bootstraps with a GTRGAMMA model, using the online service CIPRES and the supercomputer XSEDE [51]. Invariable sites (+I) were not included in the model as they are intrinsically linked to other factors such as rate categories and are unlikely to be true biologically (as all sites within a sequence are likely to have some, if negligible, mutation rate) [52,53]. This model composition is the recommended choice by the developers of RAxML [49].

To test for consistency of results, Bayesian estimation of the pygmy perch phylogeny was also conducted using PhyloBayes 4.1 [54], using a CAT substitution model and a discrete gamma distribution of site rate heterogeneity model. A Markov chain Monte Carlo chain was run for a total of 1480 cycles consisting of 91 584 tree generations until the log likelihood demonstrated a stable equilibrium. Bayesian analysis was limited to the strict dataset due to computational limitations of the software. The resultant phylogenetic trees of both methods were visualized using MEGA 7 [55], using *B. porosa* as the outgroup.

### 2.5. Species delimitation

Species were delimited using the software package Bayesian Phylogenetics and Phylogeography (BPP 3.2) [56]. Delimitation was limited to the smaller, strict dataset due to computational constraints. BPP uses a coalescent modelling method to estimate species limits in a Bayesian framework, and estimates multiple species tree hypotheses simultaneously with the delimitation to create a more robust and statistically sound analysis by providing posterior probabilities of both.

The unguided species delimitation method (analysis A11 as described within the manual) was used to simultaneously estimate both the species tree and the delimitation of the designated species, avoiding biases associated with using a fixed guide phylogeny [56,57]. As BPP can only coalesce but not split input species, all three populations of *N. vittata* were input as separate species on account of their divergent and paraphyletic nature in the maximum-likelihood phylogeny (see Results below), giving a total of 11 input species. Biologically reasonable priors for the gamma distributions of population sizes of lineages (θs) and the divergence time of the root of the phylogeny (τs) were adjusted until convergence of species delimitations were found over two runs for both incorporated algorithms (*n *= 4) to confidently provide consistent results. Other divergence time parameters were estimated using a Dirichlet prior [58, eqn (2)].

A lower number of replications was initially used for computational efficiency [10 000 burn-in + (100 sample freq * 1000 *n*sample) = 110 000 generations]. Priors were progressively altered upwards from G(2, 0) for the τs prior and G(30, 1) for the θs prior until convergence of results was found. Convergence of the runs was found using a τs prior of G(10, 10 000), with Dirichlet prior for other divergence times, and a θs prior of G(2, 100), using cleandata = 0 to account for gaps and ambiguous characters in the sequence. Once convergence was found, a final species delimitation and species tree estimation analysis was done using a larger number of generations under each algorithm [2 replicates per algorithm; 10 000 burn-in + (5 sample freq * 98 000 *n*sample) = 500 000 generations].

### 2.6. Molecular dating

Divergence time estimates were obtained using a maximum clade credibility (MCC) pipeline. The MCC method allows for the summation of thousands of phylogenetic trees into a single, consensus tree by selecting the tree containing the most common clades, rather than building a tree from each most common clade that may never have been generated in the initial analysis [59]. As ambiguous or heterozygous sites can drastically impact divergence time estimates [60,61], haplotypes were generated using repeated random haplotype sampling (RRHS), which randomly assigns a particular base to each heterozygous site within an unphased sequence [60]. A total of 3000 sets of RRHS sequences were created for divergence estimates using an edited RRHS Java command line script provided by [60], with each set of haplotype sequences analysed in a ML phylogeny using 1000 bootstraps in RAxML. The ‘best tree’ output for each RAxML run was unrooted using the R package *ape* [62] and summarized into a single MCC tree using the *consense* protocol of ExaBayes [63]. Due to issues with the formation of a polytomy within the root of the phylogeny which prevents divergence time estimation, a *B. porosa* outgroup sample (BpoHR1) was removed from the tree using the *prune* command of *ape*. This had no effect on branch lengths or topology within the rest of the phylogeny.

**Figure 2.**
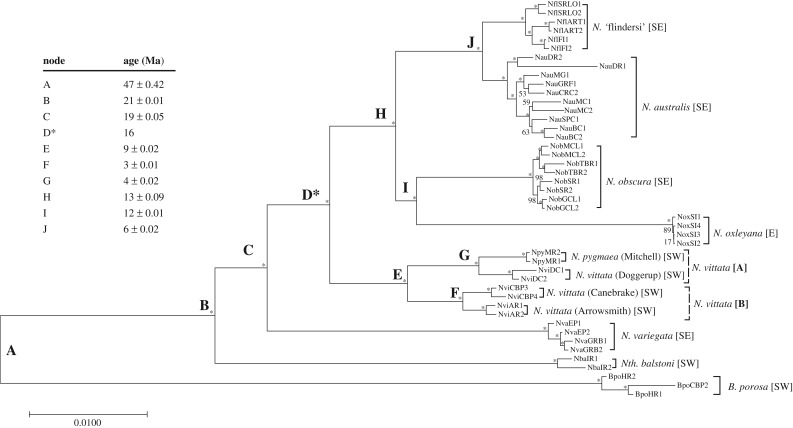
Maximum clade credibility (MCC) based on 3000 random repeated haplotypes (RRHS) of a 1 075 734 bp sequence (13 991 concatenated ddRAD loci) and divergence estimates from r8s. Bootstraps are calculated from 1000 bootstraps per RRHS summarized using the ‘best tree’ output of RAxML. All 3000 ‘best trees’ were summarized using the consense function of ExaBayes and the resultant phylogeny plotted in MEGA 7.0. All major species and population divisions had 100% bootstrap support; all bootstraps with 100% support are represented by small asterisks. Codes for individuals and localities relate to the abbreviations in table 1. Node D denotes the node used to calibrate for divergence time estimation. Divergence estimates are reported for the MCC phylogenetic tree, with associated confidence intervals from 100 subsampled RRHS trees (± s.d.).

The software r8s 1.81 was then used to estimate divergence times in the MCC phylogeny [64]. R8s estimates absolute rates of divergence times across branches of a given phylogenetic tree based on branch lengths, by using an estimation of relative rate across branches and relating this to a set age calibration of at least one given node [64]. A single calibration point was placed at the split between the eastern clade of *N. australis–N. obscura–N. oxleyana* and the western clade of *N. vittata–N. pygmaea* at 14–16 Ma. This date represents the formation of the current day Nullarbor Plain which is associated with the cessation of potential connectivity between the eastern and western freshwater fauna [27]. Divergence times for each node were estimated using a penalized-likelihood model under a truncated Newton algorithm [65], which uses a parametric branch substitution rate model with a nonparametric roughness penalty [64]. A ‘smoothing parameter’ determines the contribution of the roughness penalty aspect: a cross-validation procedure was used to determine the best value between log_10_ 0 and log_10_ 100. The optimum log_10_ smoothing parameter of 44.00, with a chi-square error of 139 049.74, was used to estimate divergence times for defined major species divisions (described in figure 2). Confidence intervals for the age estimations were calculated by sub-sampling 100 independent RRHS trees from the 3000 trees dataset. These trees were randomly selected from a pool of 2388 trees that showed matching topology to the MCC phylogenetic tree (determined using the all.equal.phylo function in *ape* [62]). Each tree was dated using the same methods as above and the distribution of node ages for all major species divisions calculated using the *profile* function of r8s. Using topologically identical trees with varying branch lengths to estimate confidence intervals is the suggested approach within the r8s manual [64]. Estimates for mutation rates across all branches, as well as the average across the phylogeny, were also calculated using r8s. Including fossil-calibrated reconstructions to estimate divergence time in this study was hampered due to the absence of known suitable fossils from pygmy perches or closely related taxa. Although evolutionarily divergent fossils exist for Centrarchidae—a freshwater family endemic to North America thought to have split from the Australian Percichthyidae over 61 million years ago [66]—applying poorly constrained fossil evidence with questionable placement in the phylogeny is not a recommended option [67,68].

### 2.7. Ancestral area reconstruction analysis

In order to statistically evaluate competing biogeographic hypotheses about multiple dispersal or vicariance events (see Discussion), we estimated ancestral areas using the R package *BioGeoBEARS* [69]. As *BioGeoBEARS* requires a time-calibrated ultrametric tree as an input file, the MCC phylogenetic tree was first collapsed down to species using the collapseTree command of the R package *phytools* [70] and calibrated using the MCC r8s divergence estimates and the chronos command of *ape.* Species were assigned to one of two biogeographic regions (East or West) with a max range of 2 (i.e. both regions) for ancestral lineages. Ancestral areas were estimated under all six available models (DEC, DIVA-LIKE and BAYAREA-LIKE, as well as their + J counterparts) and compared using the Akaike information criterion (AIC) to assess the fit of each model.

## 3. Results

### 3.1. Sequence filtering and alignment

The strict dataset (approx. 11% missing data) of 4381 ddRAD loci produced a concatenated sequence of 334 936 bp, and the relaxed dataset (approx. 30% missing data) of 13 991 ddRAD loci produced a concatenated sequence of 1 075 734 bp (table 2). Both datasets contained a large number of informative sites, with 35 856 parsimony-informative sites within the 41 067 single nucleotide polymorphisms (SNPs) of the strict dataset, and 123 252 parsimony-informative sites within the 142 476 SNPs of the relaxed dataset.

**Table 2.** Summary data for ddRAD loci and percentage of missing data (%) based on two separate filtering criteria. Strict = minimum of 40 individuals (approx. 89%) per locus; relaxed = minimum of 32 (approx. 70%) individuals per locus. PIS = parsimony-informative sites.

sequence subset	factor	strict dataset	relaxed dataset
total sequence	sequence length (bp)	334 936	1 075 734
	number of ddRAD loci	4381	13 991
	number of SNPs	41 067	142 476
	number of PIS	35 856	123 252
all individuals	mean no. of ddRAD loci	4156	12 043
	missing data (%)	5.09	13.92
ingroup only	mean no. of ddRAD loci	4278	12 459
	missing data (%)	2.34	10.95
outgroup only	mean no. of ddRAD loci	2470	6220
	missing data (%)	43.62	55.54

### 3.2. Sequence divergence analysis

For simplicity, we discuss here only the relaxed dataset sequence divergence values as both datasets demonstrated similar patterns across the group, although exact values for each pairwise comparison were slightly underestimated in the strict dataset (table 3). *Nannoperca vittata* was composed of two divergent groups (referred herein as ‘superclades’ [A] and [B]). Superclade A consisted of the Doggerup Creek lineage of *N. vittata* and *N. pygmaea*; superclade B consisted of the remaining two *N. vittata* lineages (Arrowsmith River and Canebrake Pool); these superclades were highly divergent to one another (*P* = 1.26%). Marked divergence was also observed within *N. australis* (table 3). These two taxa had the greatest levels of genetic heterogeneity, with higher intraspecific mean uncorrected (*P*) genetic distance than all other intraspecific comparisons (0.2–0.44%). In contrast, *N. oxleyana*, *N. variegata* and each individual *N. vittata* lineage (DC, AR, CBP) demonstrated remarkably low within species genetic differentiation (0.01–0.04%).

**Table 3.** Uncorrected (*P*) mean percentage genetic distance matrix of pygmy perch lineages based on two genomic datasets. The top right section of the distance matrix represents the ‘strict’ (4381 loci) dataset while the bottom left section represents the ‘relaxed’ (13 991 loci) dataset. Comparisons between a lineage and itself (*italicized,* in boxes) represent within-lineage mean genetic distance (denoted as ‘relaxed/strict’). Comparisons between a *N. vittata* superclade (A or B) and its containing lineages are not shown.

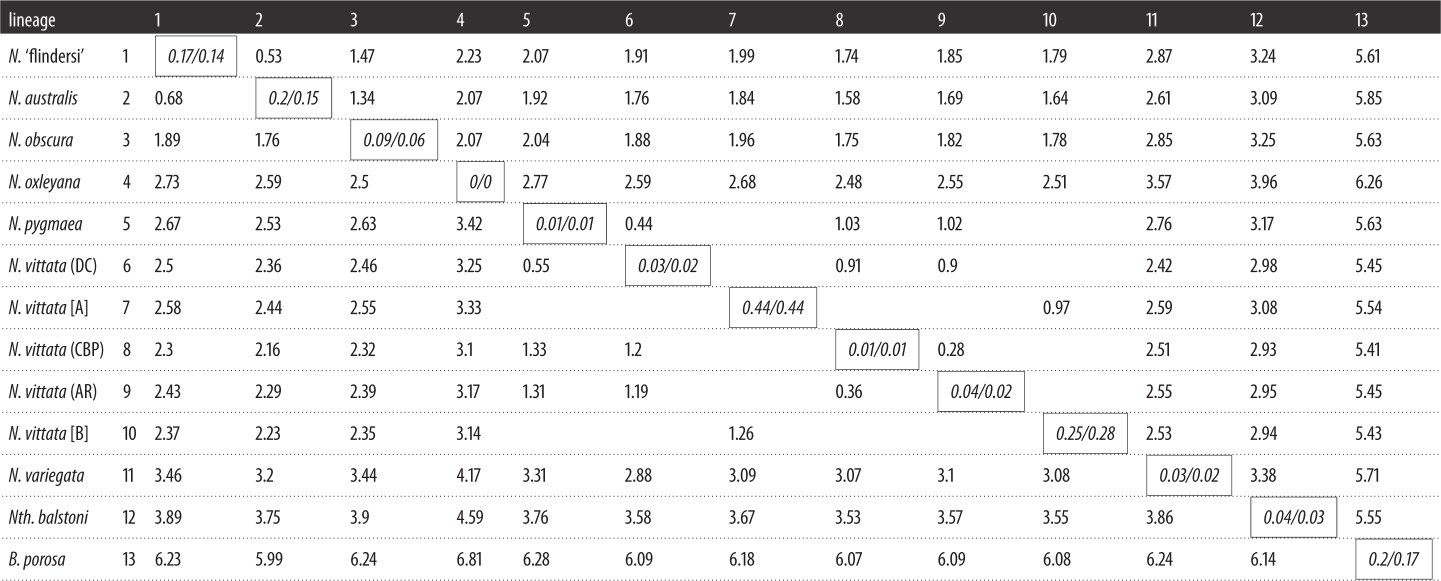

Furthermore, individual clades of *N. vittata* within each superclade showed greater genetic distance to one another (0.36–0.55%) than that found within any other species. These levels of divergence approached the genetic distance between previously suggested species, such as between *N.* ‘flindersi’ and *N. australis* (0.68%). When comparing *N. vittata* [A] and *N. vittata* [B], the divergence seen between these (1.26%) exceeds the genetic differentiation between *N.* ‘flindersi’ and *N. australis.* Finally, the eastern species *N. oxleyana* appeared as the most divergent *Nannoperca* lineage, showing the greatest pairwise genetic distance to all other pygmy perches (2.50–4.59%). Similar levels of divergence were shown for *Nth. balstoni* to all other pygmy perches (2.93–4.59%).

### 3.3. Phylogenetic analysis

Visual inspection of the ML phylogenies demonstrated no effect of missing data or loci number, with both datasets showing identical topology for all major clades within pygmy perches and similar branch lengths (electronic supplementary material, figures S1 and S2). Similarly, Bayesian analysis of the strict dataset showed no variation in topology compared to the ML phylogeny of the same dataset and returned very high nodal support (0.95-1 posterior probability for all nodes of tree; electronic supplementary material, figure S1). The MCC tree demonstrated near identical topology to the previously generated trees, albeit with significantly higher bootstrap values and a greater difference in branch lengths (figure 2). This difference is expected due to the pseudo-phasing of data and larger number of bootstraps (3000 individual trees). Topological differences were limited to changes in the position of samples NauMC1, NvaEP2 and BpoHR1 within their respective lineages. Distinctive branch length variations were observed in Darby River *N. australis* samples (NauDR), within individual *N. vittata* locality groups (NviAR, NviDC and NviCBP), within *Nth. balstoni* and the outgroup *B. porosa.* Bootstrap support varied slightly across datasets, with the strict dataset giving higher support for some nodes (within *N. australis*, *N.* ‘flindersi’ and *N. variegata*), but lower support for others (within and between *N. obscura* and *N. oxleyana*). This most likely reflects a bias towards *N. australis*-specific loci in the strict dataset due to the higher number of samples for this taxon.

Four of the five species from eastern Australia (*N. australis*, *N.* ‘flindersi’, *N. obscura* and *N. oxleyana*) were reciprocally monophyletic. This clade was sister to a western clade comprised of *N. vittata* (including all putative cryptic forms within this species) and *N. pygmaea*. The combined eastern and western clade was sister to an eastern species, *N. variegata*, and these together were sister to a western species, *Nth. balstoni*. Additionally, the phylogeny included the distinct separation of *N.* ‘flindersi’ from its sister taxon *N. australis* and divergence of *N. pygmaea* and clades of *N. vittata*.

Finer scale population-level patterns could also be observed within several taxa, with *N.* ‘flindersi’ separating into one Victorian (Snowy River Lagoon, Orbost) and one Tasmanian (Flinders Island + Anson River) clade. Similarly, *N. australis* separated into three clades: one composed of Murray–Darling Basin individuals, one of fish from the southeast coast and one of a distinct population at Darby River. Shallower phylogeographic patterns were also observed within *N. obscura*, with geographical clusters of populations forming distinct clades.

### 3.4. Species delimitation

Coalescent modelling of species delimitation resulted in a total of 11 species within the phylogeny (posterior probability = 1 for all runs), with each clade within *N. vittata* being recognized as an independent species. All previously described or suggested species were similarly recognized as independent of one another, including *N.* ‘flindersi’ and *N. pygmaea*. The species tree output by BPP matched the topology of all ML and Bayesian trees and the MCC tree.

### 3.5. Molecular dating

Divergence time estimates from r8s revealed that pygmy perches are an ancient lineage, with the root of the group estimated at 20 (±0.01) Ma (figure 2). This time of origin is similar to other ‘ancient’ teleosts from the northern hemisphere [71,72]. Each inferred east–west divergence had a different estimated age within the pygmy perch radiation. Most lineages within the major eastern clade showed comparatively recent divergences, except for *N. oxleyana* which showed an older divergence time of 12 (±0.01) Ma from *N. obscura* (node I), reflected by the high genetic distance to all other pygmy perches. More closely related species had relatively young ages such as 3–4 Ma (nodes F and G) within the *vittata* clade and 6 (±0.02) Ma between *N. australis* and *N.* ‘flindersi’ (node J). All times of divergences were found to be within or close to the ranges proposed by Unmack *et al.* [27]. All estimations of node ages were highly consistent, as indicated by low standard deviation of all node estimations using 100 RRHS trees (electronic supplementary material, table S1; figure 2). This is expected as individual RRHS trees do not excessively vary in branch lengths and indicates that even pseudo-phasing of the data can produce consistent results.

Estimations of rate variation across sequences had a mean rate of 9.67 × 10^−4^ (±4.81 × 10^−7^ standard deviation) substitutions per site per million years. Very small differences in rate variation were seen across lineages, as highlighted by the low standard deviation of total rate variation.

### 3.6 Ancestral area reconstruction

**Figure 3.**
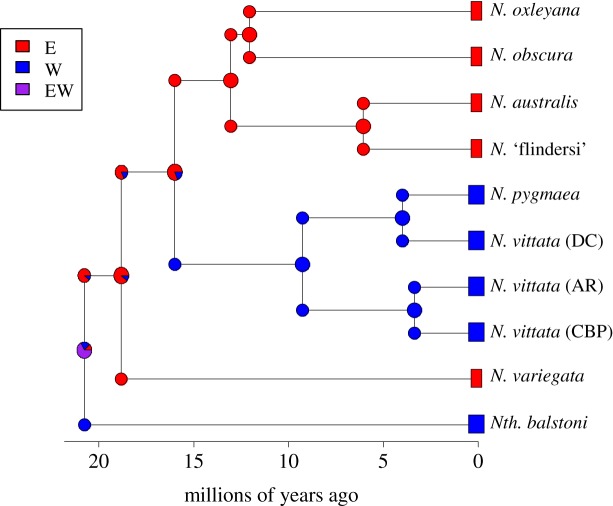
The most supported ancestral area reconstruction model (DIVALIKE+J), estimated within the R package *BioGeoBEARS*. Colours denote geographical range of lineages, with pie charts representing the relative probability of geographical range of ancestors. Lineages were collapsed down to the species level and the phylogeny time calibrated based on the MCC divergence estimates from r8s.

Evaluation of the six potential biogeographic models within *BioGeoBEARS* using AIC suggested that the DIVALIKE+J model was best representative of the data (table 4, figure 3). This model demonstrated low probability of dispersal or extinction events (*d* and *e *= 1 × 10^−12^) but showed an effect of a founder event (*J *= 0.0972). Furthermore, nearly all ancestral lineages demonstrated a likely eastern geographical range (excluding the ancestor of the two pygmy perch genera). These results suggest that a founder event (such as a rare, long-distance dispersal event) from the east was a likely driving factor in the colonization of the major western clade containing the *N. vittata* species complex (including *N. pygmaea*).

**Table 4.** Statistical evaluation of biogeographic models implemented in *BioGeoBEARS.* Extant species were assigned to an eastern or western geographical range with a maximum range of 2 (i.e. ancestors could occupy both areas) for all analyses, with the accuracy of each model assessed and compared using the Akaike information criterion (AIC).

model	log likelihood	dispersal (*d*)	extinction (*e*)	founder event (*J*)	AIC
DEC	−5.38	1.00 × 10^−12^	1.00 × 10^−12^	0	14.75
DEC+J	−4.36	3.50 × 10^−12^	1.60 × 10^−10^	0.1	14.71
DIVALIKE	−6.14	0.0083	1.00 × 10^−12^	0	16.27
DIVALIKE+J	−3.96	1.00 × 10^−12^	1.00 × 10^−12^	0.097	13.93
BAYAREALIKE	−9.29	1.00 × 10^−12^	0.029	0	22.58
BAYAREALIKE+J	−4.96	1.00 × 10^−12^	1.00 × 10^−12^	0.13	15.92

## 4. Discussion

We performed a genome-wide study using phylogenomic and species delimitation methods to clarify evolutionary and biogeographic history and to assess cryptic diversification in all known lineages of pygmy perches. We confirmed the biological relevance of previously inferred phylogenies and recently suggested species. Additionally, with this much larger dataset, we provided greater phylogenetic resolution and discovered extra cryptic species. Our findings reveal complex biogeographic patterns in southern Australia and point to the importance of in-depth taxonomic analyses for appropriate conservation management of threatened biodiversity.

### 4.1. Phylogenomics of pygmy perches

Our phylogenetic trees corroborate the combined tree presented by Unmack *et al.* [27] and went further by providing substantially improved phylogenetic resolution. There was a major improvement in bootstrap support for all nodes in the genomic phylogeny, highlighting the ability of ddRAD to avoid locus-specific biases such as mitochondrial introgression, which may have obscured some findings of the previous study. These phylogenetic trees enabled the resolution of conflicting nodes across different markers from Unmack *et al.* [27]: all phylogenies here indicate that *N. obscura* is sister to *N. oxleyana* and not to *N. australis*, and that *N. variegata* is the first branching lineage within *Nannoperca*. Furthermore, the paraphyletic nature of *N. vittata* further suggests that the taxonomy of the species is currently unresolved. Of particular note is the high level of divergence observed between individual, geographically isolated lineages within *N. vittata* reflected in both the genetic distance and divergence estimates results (table 3, figure 2). The level of divergence between these *N. vittata* lineages is similar to that between *N. vittata* and *N. pygmaea*, possibly indicating cryptic species within *N. vittata* (see below). This study also enabled a much greater resolution of intraspecific population structure within *N. australis* and *N.* ‘flindersi’ than suggested by prior studies (e.g. [36]).

A species delimitation framework, in combination with other qualitative genetic analyses, supported the proposed *N. pygmaea*, which was previously only based on morphological assessments and limited allozyme data [27,73]. We also revealed two new cryptic lineages within *N. vittata*. It is recommended that a more thorough analysis of *N. vittata* should be conducted in accordance with an integrative taxonomic approach [74–76] before taxonomic changes are implemented and used in conservation management [77,78]. Assessing all known populations of *N. vittata*, with more comprehensive sampling than achieved here, may reveal the full geographical range and ecological nature of the delimited species. Additional research focused on morphological and ecological divergences is important for establishing species boundaries which would help resolve their taxonomy. Clearly, further taxonomic studies of pygmy perches are needed to better inform their conservation management, particularly for those lineages without formal description and legislative protection such as *N.* ‘flindersi’. An understanding of species limits and local adaptation would provide a framework for targeted conservation actions in pygmy perches, such as genetic rescue [79].

While species delimitation provides a statistical framework for testing hypotheses of species identity, there are limitations in the outcomes. Simulations have suggested that BPP has a tendency to exaggerate the number of species within a phylogeny, providing high support for divergent lineages which are not biologically true species [80]. Despite this criticism, a solely genetic basis for species description has rarely (if ever) been used [81]. Taxonomic changes require a complement of various forms of analyses, including morphological, ecological and behavioural data [12,76] to infer species identity and reproductive isolation. Thus, while we do not present our delimitations as fully putative species, we conservatively suggest that those identified may possibly reflect truly cryptic species.

### 4.2. Biogeographic interpretation

**Figure 4.**
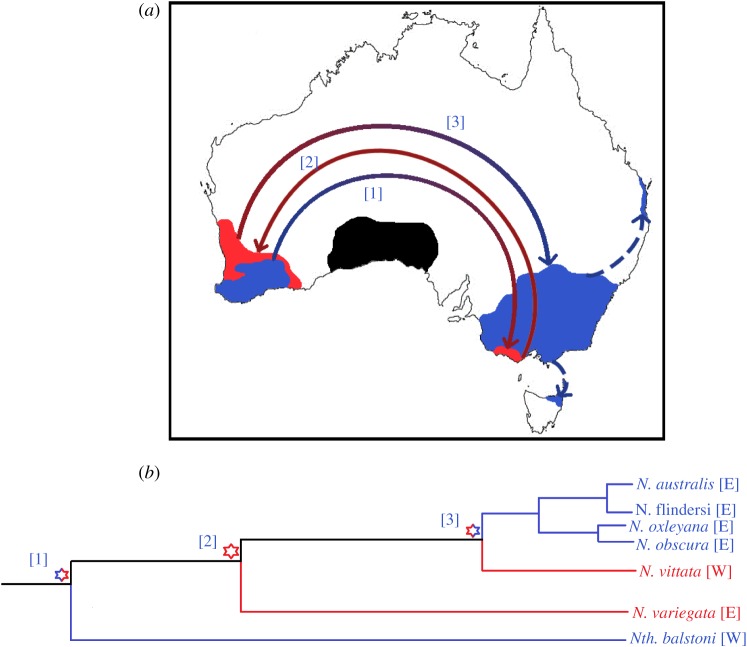
Hypothetical model of the Multiple Invasion Hypothesis and the biogeography of pygmy perches. (*a*) Graphical representation of Multiple Invasion (under the maximum number of theoretical migrations) as a possible mechanism for the biogeography of pygmy perches. The position of the Nullarbor Plain is demonstrated in black. Migrations of pygmy perches are indicated by the directionality and colour of the arrows, with contemporary distributions demonstrated by the filled regions. Dashed arrows represent secondary migrations (into *N. oxleyana* and *N.* ‘flindersi’) without crossing the continent. (*b*) Phylogenetic diagram of geographical divergences in pygmy perches under Multiple Invasion. Nodes representing migration events are denoted within the phylogeny by asterisks, with the numbers corresponding to a particular migration on the map. Divergence [3] represents the last possible migration event before the arrival of the Nullarbor Plain as a barrier to dispersal.

Our study provides support for multiple east–west movement events across southern Australia involving both older (i.e. *Nth. balstoni* and *N. variegata*), as well as younger pygmy perch lineages. The ancestral area reconstruction analysis did not support a simple model of vicariant divergence of eastern and western pygmy perches as the result of the Nullarbor Plain. Instead, the most supported model suggested a founder event from an eastern ancestor into the west (the predecessor of the *N. vittata* species group). Given that the climatic and geological changes associated with the formation of the Nullarbor Plain were likely gradual, it is possible that a peripheral population of the eastern ancestor became isolated during the Miocene. Alterations to hydrology and geology, such as river capture, may have disconnected this peripheral population from the rest of the species range, causing it to divert westward and found the western pygmy perch group. This is particularly relevant for freshwater species which inhabit dendritic systems as geological and climatic changes can significantly alter hydrological connections, thereby having significant impacts on phylogeographic structure and interpretation [82]. Thus, we suggest that range expansion, followed by subsequent isolation due to a vicariant barrier, is likely the major mechanism driving this geographical separation. Nonetheless, we assume that the formation of the Nullarbor Plain was still pivotal to the complete separation between younger western (*N. vittata*) from younger eastern (*N. australis*, *N. obscura* and *N. oxleyana*) lineages. This scenario is similar to that proposed by Unmack *et al.* [27], who suggested that the lack of a singular east–west split of pygmy perches was indicative of multiple migrations across central southern Australia prior to the formation of the Nullarbor Plain (deemed the ‘Multiple Invasion Hypothesis’; figure 4). While projections of historical climate suggest that the hydrology of southern Australia may have been suitable for the migration, little support for multiple migrations has been found in other aquatic taxa [2,29,32]. Of these, many are more dispersive than pygmy perches, suggesting they should likewise have been able to migrate across southern Australia multiple times. While this may seem counterintuitive, little is known about the historic ecology of aquatic biota to propose a mechanism or reason for this disparity. The historically widespread distributions of *N. vittata* and *N. australis* have been suggested to predispose them to being tolerant to a range of habitats [27]: thus, it is hypothetically possible that pygmy perches were able to tolerate intermediate habitats between the east and west. Additionally, historical metapopulation dynamics suggested for pygmy perches may have allowed them to respond to temporally unfavourable habitats [40].

**Figure 5.**
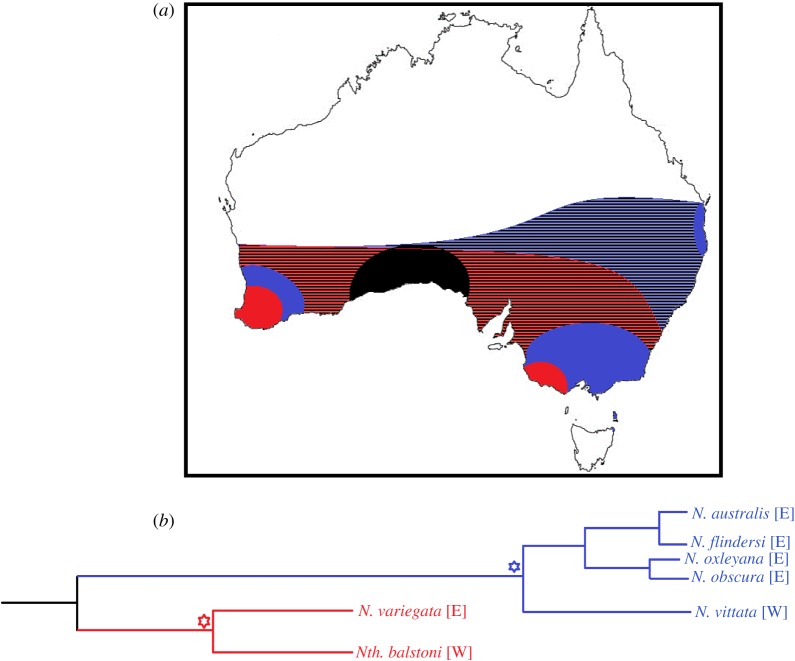
Hypothetical model of geographical paralogy and the biogeography of pygmy perches. (*a*) Graphical representation of geographical paralogy as a possible mechanism for the biogeography of pygmy perches. The position of the Nullarbor Plain is demonstrated in black. Speculative historic ranges of the two ancestral lineages required for geographical paralogy to occur are demonstrated by the striped regions, with contemporary distributions demonstrated by the filled regions. (*b*) Phylogenetic diagram of geographical divergences in pygmy perches under geographical paralogy. Nodes representing E/W divergences are denoted within the phylogeny by asterisks, with the second (blue) divergence representing the arrival of the Nullarbor Plain as a barrier to dispersal.

However, the Multiple Invasion Hypothesis for pygmy perches was later criticized, with Ladiges *et al.* [83] instead proposing that geographical paralogy may explain the lack of a singular east–west split. Geographical paralogy is when two (or more) independent but closely related lineages experience a biogeographic split at the same time (figure 5). In the case of pygmy perches, the geographical split of one lineage containing the ancestor of *Nth. balstoni* and *N. variegata*, and one lineage containing the ancestor of *N. australis*, *N. obscura*, *N. oxleyana* and *N. vittata*, would produce a similar phylogenetic pattern to that shown here. These independent divergence events could have been vicariantly caused by the formation of the Nullarbor Plain, or could reflect independent splitting events (both in mechanism and timing). There are unfortunately no known fossil records of pygmy perches across southern Australia to demonstrate the necessary ubiquity of pygmy perches prior to these splits [84].

The Multiple Invasion Hypothesis is instead more strongly supported than geographical paralogy by the current study. This is based on the lack of a sister relationship between *N. variegata* and *Nth. balstoni* required for geographical paralogy (electronic supplementary material, figures S1 and S2; figures 2 and 5). This lack of sister group relationships would likely only be incorrect if there are unaccountable artefacts from extinction of lineages or difficulties in detecting relationships in anciently diverged lineages. Additionally, the ancestral area reconstruction modelling did not suggest a simple vicariant separation of eastern and western pygmy perches from a widely distributed ancestor, but instead a founder event (e.g. long-distance dispersal) from the east to the west (figure 3). While there are significant limitations and assumptions with historical biogeographic analysis, alternative models that included many widespread ancestors were not as well supported by the data (table 4).

### 4.3. Biodiversity hotspot in southwestern Australia

The highly divergent nature of populations in endemic species (*N. vittata* spp. and *Nth. balstoni*) of southwestern Western Australia (figure 2) indicates that this region is a significant driver of evolution and speciation. This is consistent with other studies that showed high levels of endemism and species diversity within the region, leading to its internationally and nationally recognized status as a biodiversity hotspot [28,29]. Our findings reinforce the high conservation value of the region.

The high levels of *in situ* speciation in southwestern Australia have often been linked to historical climate changes [29,30]. The drivers of population diversification within pygmy perches probably relate to allopatric isolation as a result of fragmentation of wetter regions, with all species of western pygmy perches limited to areas of higher annual rainfall (greater than 600 mm) [29]. Allopatric speciation has been noted throughout southwest Western Australia for a range of taxonomic groups with limited dispersal capabilities, including many invertebrate groups (e.g. spiders, isopods and crayfishes) and some frog species [29,85,86].

It is likely that a combination of long-term isolation associated with formation of the Nullarbor Plain, as well as *in situ* speciation within southwest Western Australia, are major factors accounting for the diversification and evolution of pygmy perches. Further assessments focusing on biogeographic history are required to elucidate the complexity of the pygmy perch phylogeny. In this regard, species distribution modelling (SDM) appears as a robust approach to addressing competing biogeographic theories when combined with statistical phylogeographic methods [87,88]. For pygmy perches, SDM could be applied to southwestern Australia to determine what role environmental factors played in driving the divergence of *N. vittata* lineages.

### 4.4. Conservation concerns

The identification of cryptic species has profound implications for their conservation management, particularly for legislation. While *N. vittata* is currently unlisted within the IUCN Red List, our results suggest that *N. vittata* is potentially three independent species, each with a small number of narrow endemic populations that are at risk of losing genetic diversity and local extirpation. Thus, a thorough assessment of *N. vittata* throughout their currently recognized range and associated species descriptions are required to fully elucidate the taxonomic intricacies within this lineage and to incorporate these into conservation management practices.

Our study also identified intraspecific lineages that are not yet divergent enough to be considered as different species (as opposed to the divergent lineages within *N. vittata*). Some of these lineages correlated with previously identified ESUs [89], such as in *N. obscura* [42]*.* Additional intraspecific genetic structure in *N.* ‘flindersi’ and *N. obscura* suggests that conservation managers should recognize different conservation units. This includes three geographically isolated lineages of *N.* ‘flindersi’. Given the isolation of these lineages by unpassable saltwater barriers (Bass Strait) and environmental differences between the regions [90,91], they may be adaptively divergent and are perhaps precursors of incipient species. A thorough assessment of population structure using genome wide data in each region is required for improving and defining appropriate units for management within species.

Both the taxonomic issues demonstrated by this study, as well as the low genetic diversity and dispersal capabilities of many pygmy perches [33,37,39,40,42], raise concerns for their conservation management under future climate change. Their low diversity potentially reduces their capacity to adapt to changing environments, and their low dispersal capabilities inhibit them from easily moving to more favourable environments. In addition, low genetic variation and human-induced adaptive divergence in habitat fragments threaten the species in the Murray–Darling Basin [39] (see also [40]). Genetic-based captive breeding and restoration efforts have already been required for *N. australis* and *N. obscura* in the lower Murray River [37,92], and local extirpations in various parts of the Murray–Darling Basin are already recorded [38,40]. Our novel phylogeographic findings are expected to inform future genetic-based breeding programmes in pygmy perches, particularly for *N. vittata* spp., as such programmes should take into account the evolutionary history and historical demography of threatened lineages to maximize their success [37].

We used genomics to identify and characterize biodiversity patterns across southern Australia and within the pygmy perches. Specifically, we aimed to improve our understanding of southern Australian biogeography and the taxonomy of pygmy perches, with associated implications for conservation. Using a powerful dataset, we have resolved previously uncertain phylogenetic relationships in pygmy perches, identified and confirmed additional cryptic species within *N. vittata* through a species delimitation framework, and estimated divergence times for the pygmy perch phylogeny using a ddRAD molecular clock. These findings have strengthened our understanding of biological concepts such as southern Australian biogeography, the need for further taxonomic research into pygmy perches and important conservation suggestions. The indication of high levels of cryptic speciation within southwest Western Australia further corroborates its high conservation importance and biodiversity hotspot identity, while the identification of independent species suggests modification for current conservation statuses and management.

## Supplementary Material

Phylogenomics and species delimitation of pygmy perches (Teleostei: Percichthyidae): implications for biogeography, taxonomy and conservation

## Data accessibility

The concatenated sequence input files under both alignment conditions are available in PHYLIP format in Dryad (http://dx.doi.org/10.5061/dryad.3d6g1) [93].
