# Supplementary material for: Phylogenomic history of enigmatic pygmy perches: implications for biogeography, taxonomy and conservation
Source: R Soc Open Sci. 2018 Jun 13;5(6):172125. doi: 10.1098/rsos.172125 (PMC6030323; doi:10.1098/rsos.172125)
Supplement: Phylogenomics and species delimitation of pygmy perches (Teleostei: Percichthyidae): implications for biogeography, taxonomy and conservation [file rsos172125supp1.docx]

Supplementary Material for the paper:

**Phylogenomic history of enigmatic pygmy perches: implications for biogeography, taxonomy and conservation**

**Table S1:** Divergence estimates and mutation rates for all major nodes of the pygmy perch phylogeny using r8s and constraining the node *NP* with the formation of the Nullarbor Plain as a barrier to dispersal 14-16 Ma. Nodes are listed and shown within Figure 2 for simplicity, with E/W divergence events listed here (*mixed* specifies downstream lineages containing both geographies). Divergences were estimated using the penalized-likelihood (PL) method under the truncated Newton [64] algorithm. Rates are estimated in substitutions per site per million years, with the estimated rate for the entire tree and the local rate done for only the relevant branch point. Confidence intervals of estimations were estimated using 100 independent RRHS trees, with the means and standard deviation of nodes reported here. CI = confidence interval.

| **Node** | Geography of resultant branches | MCC estimated age (Ma) | Mean RRHS estimated age | RRHS standard deviation of estimated ages | Estimated rate | Local rate | Mean divergence estimate (Ma) from Unmack *et al.* (2011) [95% CI] |
| --- | --- | --- | --- | --- | --- | --- | --- |
| Root | West/Mixed | 46.71 | - | - | -- | -- | 41.8 [27.7-56.3] |
| BalRoot | West/Mixed | 20.76 | 22.97 | 0.09 | 9.66E-04 | 2.80E-03 | 26.9 [20.8-34.1] |
| VarRoot | East/Mixed | 18.81 | 20.08 | 0.05 | 9.67E-04 | 2.26E-03 | 21.3 [17.0-26.6] |
| NP | East/West | 16.00 | 16.00 | 0.00 | 9.67E-04 | 1.89E-03 | 15.9 [14.1-19.1] |
| VitPair | West | 9.27 | 9.07 | 0.02 | 9.67E-04 | 9.80E-04 | 9.6 [6.3-13.0] |
| VitB | West | 3.35 | 3.22 | 0.01 | 9.67E-04 | 7.96E-04 | - |
| VitAPyg | West | 4.00 | 3.87 | 0.01 | 9.67E-04 | 1.15E-03 | - |
| EasRoot | East | 13.04 | 12.87 | 0.01 | 9.67E-04 | 1.89E-03 | 12.3 [9.4-15.6] |
| NoxRoot | East | 12.06 | 11.87 | 0.01 | 9.67E-04 | 1.80E-03 | 11.2 [8.2-14.6] |
| AusRoot | East | 6.07 | 5.84 | 0.02 | 9.67E-04 | 1.06E-03 | 4.05 [2.3-5.9] |

**
Figure S1:** Phylogenetic tree of pygmy perches based on the strict dataset of 4,381 ddRAD loci, rooted using *Bostockia porosa*. Branch lengths were estimated using RAxML under the GTRGAMMA model of evolution for maximum likelihood (ML) and PhyloBayes under the CAT model of evolution for Bayesian analysis. ML bootstrap values are based on 1,000 replicates (left) and Bayesian posterior probabilities estimated every 10 samples with the first 200 trees discarded as burn-in (right). Node support values are shown for all major species and population divisions. Lineages in parentheses represent species groups used for further species delimitation analysis in BPP, with their geographic location specified in the tree. Codes for individuals and localities relate to the abbreviations in Table 1.


**Figure S2:** Maximum likelihood tree of pygmy perches based on the relaxed dataset of 13,991 ddRAD loci, rooted using *Bostockia porosa*. Branch lengths were estimated using RAxML under the GTRGAMMA model of evolution. Bootstrap values are based on 1,000 replicates, with values shown for all major species and population divisions. Lineages in parentheses represent species groups used for further species delimitation analysis in BPP, with their geographic location specified in the tree. Codes for individuals and localities relate to the abbreviations in Table 1.
